# Primary cilia modulate TLR4-mediated inflammatory responses in hippocampal neurons

**DOI:** 10.1186/s12974-017-0958-7

**Published:** 2017-09-19

**Authors:** Hyunjung Baek, Hyo Jung Shin, Jwa-Jin Kim, Nara Shin, Sena Kim, Min-Hee Yi, Enji Zhang, Jinpyo Hong, Joon Won Kang, Yonghyun Kim, Cuk-Seong Kim, Dong Woon Kim

**Affiliations:** 1 Department of Anatomy, Department of Medical Science, Brain Research Institute, Chungnam National University School of Medicine, Daejeon, 35015 Republic of Korea; 2 Department of Pediatrics, Chungnam National University Hospital, Chungnam National University School of Medicine, Daejeon, 35015 Republic of Korea; 3 Department of Anesthesia and Pain Medicine, Chungnam National University Hospital, Daejeon, 35015 Republic of Korea; 4 Department of Plastic Surgery, Chungnam National University Hospital, Daejeon, 35015 Republic of Korea; 5 Department of Neuroscience and Cell Biology, The University of Texas Medical Branch School of Medicine, 301 University Boulevard, Galveston, TX 77555 USA; 6 Department of Anesthesia Medicine, Yanbian University Hospital, Yanbian, 133000 China; 7 Department of Chemical and Biological Engineering, The University of Alabama, Tuscaloosa, AL 35487 USA; 8 Department of Physiology, Department of Medical Science, Chungnam National University School of Medicine, Daejeon, 35015 Republic of Korea; 9 LES Corporation Inc., Gung-Dong 465-16, Yuseong-Gu, Daejeon, 305-335 Republic of Korea

**Keywords:** Primary cilia, Hippocampus, NFκb, TLR4, Neuroinflammation

## Abstract

**Background:**

The primary cilium is an organelle that can act as a master regulator of cellular signaling. Despite the presence of primary cilia in hippocampal neurons, their function is not fully understood. Recent studies have demonstrated that the primary cilium influences interleukin (IL)-1β-induced NF-κB signaling, ultimately mediating the inflammatory response. We, therefore, investigated ciliary function and NF-κB signaling in lipopolysaccharide (LPS)-induced neuroinflammation in conjunction with ciliary length analysis.

**Methods:**

Since TLR4/NF-κB signaling is a well-known inflammatory pathway, we measured ciliary length and inflammatory mediators in wild type (WT) and TLR4^−/−^ mice injected with LPS. Next, to exclude the effects of microglial TLR4, we examined the ciliary length, ciliary components, inflammatory cytokine, and mediators in HT22 hippocampal neuronal cells.

**Results:**

Primary ciliary length decreased in hippocampal pyramidal neurons after intracerebroventricular injection of LPS in WT mice, whereas it increased in TLR4^−/−^ mice. LPS treatment decreased primary ciliary length, activated NF-κB signaling, and increased Cox2 and iNOS levels in HT22 hippocampal neurons. In contrast, silencing Kif3a, a key protein component of cilia, increased ARL13B ciliary protein levels and suppressed NF-κB signaling and expression of inflammatory mediators.

**Conclusions:**

These data suggest that LPS-induced NF-κB signaling and inflammatory mediator expression are modulated by cilia and that the blockade of primary cilium formation by Kif3a siRNA regulates TLR4-induced NF-κB signaling. We propose that primary cilia are critical for regulating NF-κB signaling events in neuroinflammation and in the innate immune response.

**Electronic supplementary material:**

The online version of this article (10.1186/s12974-017-0958-7) contains supplementary material, which is available to authorized users.

## Background

Cilia are conserved, microtubule-based organelles that grow from basal bodies (centrosome-derived structures) and extend from the cell surface [1]. Cilia play crucial roles in vertebrate development and human genetic disease. They have been hypothesized to act as “antennae” during signal detection, which, in many contexts, has proven to be the case [2]. Many genetic disorder ciliopathies are involved in the loss or impairment of the primary cilia. Recent studies, however, indicate that specialized ciliary compartments often influence signal transduction downstream of the initial stimulus, but upstream of the transcriptional response. Indeed, there are now established functions for primary cilia in left-right determination, mechanosensation, and regulation of Sonic hedgehog (Shh) [3, 4], Wnt [5], insulin growth factor (IGF), and transforming growth factor (TGF) signaling [6].

Neurons and astrocytes have been found to have a non-motile primary cilium [7]. Primary cilia have been implicated in neuronal signaling and in the central control of appetite [8, 9]. Despite the widespread presence of primary cilia in hippocampal neurons, their function remains unknown [10, 11]. Recent studies demonstrated that primary cilia contribute to IL-1β-induced NF-κB signaling by regulating IKK activity and mediating the inflammatory response [12, 13]. Additionally, in articular chondrocytes, IL-1β-induced primary cilia elongation is associated with a transient increase in HIF-2α expression, which suggest that the primary cilium regulates HIF signaling during inflammation [14]. It is also well known that changes in ciliary traffic and content affect ciliary length. Changes in ciliary length are thus indicative of altered function, as observed in Wnt and Sonic hedgehog signaling [15, 16]. The function of biogenesis of primary cilia was investigated with different stages of ciliogenesis [early (intracellular), late (extracellular)] [17]. Ciliary length is also hypothesized to be linked to the inflammatory response in chondrocytes [12, 13]. Therefore, we utilized ciliary length analysis to investigate the role of primary cilia in NF-κB signaling in LPS-induced neuroinflammation in vitro*,* using HT22 hippocampal neurons, and in vivo using a wild type and TLR4^−/−^ mice since TLR4/NF-κB signaling is a well-known inflammatory pathway.

## Methods

### Experimental animals and lesions

We used male 8–10-week-old C57BL/6 mice purchased from SamtakoBioKorea (Osan, Korea) and C57BL/10ScNJ toll-like receptor (TLR4) knockout (TLR4^−/−^) mice were purchased from the Jackson Laboratories (Bar Harbor, ME, USA). All animal-related procedures were conducted in accordance with the guidelines of the Institutional Animal Care and Use Committee of Chungnam National University (CNU-00781) and were consistent with the ethical guidelines of the National Institutes of Health. LPS was prepared as a stock solution at 10 mg/ml in sterile 0.1 M PBS. LPS was injected at right lateral cerebral ventricle (anteroposterior, − 0.4 mm; mediolateral, 1 mm; dorsoventral, − 2.3 mm relative to bregma) using a 50-μl Hamilton microsyringe fitted with a 26-gauge needle inserted to a depth of 2.4 mm (5 μg/5 μl in PBS, i.c.v.). Control mice received an equal volume of saline. LPS-injected animals (*n* = 4–5 per group) and saline-injected control animals (*n* = 5 per group) were allocated. At 1 and 3 days after LPS or saline injection, mice were anesthetized with sodium pentobarbital (50 mg/kg i.p.), and perfused transcardially with heparinized PBS, followed by perfusion with 4% paraformaldehyde in PBS. Their brains were removed, immersed in the same fixative for 4 h, and then cryoprotected in a 30% sucrose solution. They were embedded in tissue freezing medium and then frozen rapidly in 2-methyl butane precooled to its freezing point with liquid nitrogen. Frozen coronal sections (35 μm thick) were obtained using a Leica cryostat.

### Immuonohistochemistry and immunofluorescence

Parallel free-floating sections were subjected to endogenous peroxidase blocking with 1% H_2_O_2_ in PBS, followed by treatment with blocking buffer (1% fetal bovine serum (FBS) in PBS and 0.3% Triton X-100 for 30 min) and incubation with primary anti-acetylcholine III (ACIII, 1:500, #sc-588, Santa Cruz, CA) overnight. Immunohistochemical staining of the tissue sections were performed using the avidin–biotin peroxidase complex (ABC) method described previously [11, 18]. HT22 hippocampal cells were immunoreacted for ADP ribosylation factor like GTPase 13B (ARL13B, 1:1000, #17711-1-AP, Proteintech). All immunoreactions were incubated with Cy3-conjugated anti-rabbit secondary antibody and counterstained with DAPI.

### Cell culture

HT22 cells were maintained in Dulbecco’s Modified Eagle’s Medium containing 10% fetal bovine serum (WelGENE, Daegu, Korea) and 1% antibiotic solution (Gibco BRL-Life Technologies) in a humidified incubator with 5% CO_2_ in air at 37 °C. For neuronal inflammation experiments, cells were seeded in 60 mm cell culture dishes (Corning, NY, USA) 24 h before treatment. Cells were treated with 100 ng/ml lipopolysaccharides (LPS) from *Escherichia coli* 026:B6. Cells were processed for immunocytochemical and immunoblot analysis for indicated times after LPS treatment.

### Transfection with small interference RNA

For transfection with small interference RNA (siRNA), HT22 hippocampal cells were trypsinized and seeded in a 60-mm dish at 50% confluence. si_Kif3a (sense sequence: AGCUGUGAAUUAAGGAAUGGGCAGA, NM_008443) was purchased from Integrated DNA Technologies, Inc., and resuspended with DEPC water to protect from nuclease degradation. The individual siRNAs, oligofectamine, and Opti-MEM (Invitrogen, MD) were mixed and incubated at room temperature for 20 min. siRNA–oligofectamine complexes were incubated with the cells for 5 h in Opti-MEM with 10% FBS (siRNA at 20 nM final concentration). siRNA oligofectamine complexes were removed, and the cells were placed in growth media for 24 h. At 1, 6, and 24 h after LPS (100 ng/ml) treatment at the indicated concentrations, the cells were processed for immunoblotting.

### Western blotting

Hippocampi from LPS-treated (1 and 3 days) control mice and TLR4 knockout mice were dissected and homogenized in lysis buffer. After centrifugation, protein concentrations were determined in supernatants using Micro BCA Protein Assay kits; bovine serum albumin was used as a standard (Pierce Chemical, Rockford, IL, USA). Cultured HT22 cells were collected by scraping, and the pellet was solubilized in lysis buffer using PRO-PREP reagent (Intron Biotechnology, Sungnam, Korea) with a protease inhibitor cocktail. Following normalization of protein content in each sample, 30 μg of the total cellular fraction of each sample was separated by 10 or 12% SDS-polyacrylamide gel electrophoresis (PAGE) and transblotted onto nitrocellulose membranes. The blot was probed with primary antibodies: P-p65 (1:1000, #3033, Cell Signaling), IκBα (1:300, #sc-371, Santa Cruz), Cyclooxygenase 2 (Cox2, 1:500, #sc-166,475, Santa Cruz), inducible nitric oxide synthase (iNOS, 1:500, #n32030, Transduction Laboratories), and β-actin (1:500, #2965, Cell Signaling). The immune complexes were identified using an enhanced chemiluminescence (ECL) detection system (Amersham).

### Cilia length measurement

A Leica (TCS SP8) confocal microscopy was used to create maximum projection of confocal z-stacks from which cilia length was measured using ImageJ software. Different mounted preparations were used to capture five fields of cells at × 63 magnification giving data for more 100 cilia per subgroup. Confocal *z* maximum projections were also used to assess cilia prevalence and DAPI nuclear staining.

### Statistical analysis

Differences of cilia length were assessed by Chi-squared tests. Statistical significance between multiple groups was compared by one-way analysis of variance (ANOVA) followed by an appropriate multiple comparison test (post Bonferroni test). Two group analyses were performed using the Student’s *t* test. *P* values < 0.05 were considered statistically significant. All statistical analyses were performed using GraphPad Prism4 software (GraphPad Software Inc.).

## Results

### LPS decreases primary cilia length in hippocampal pyramidal neurons in wild type mice, but has the opposite effect in TLR4^−/−^ mice

**Fig. 1:**
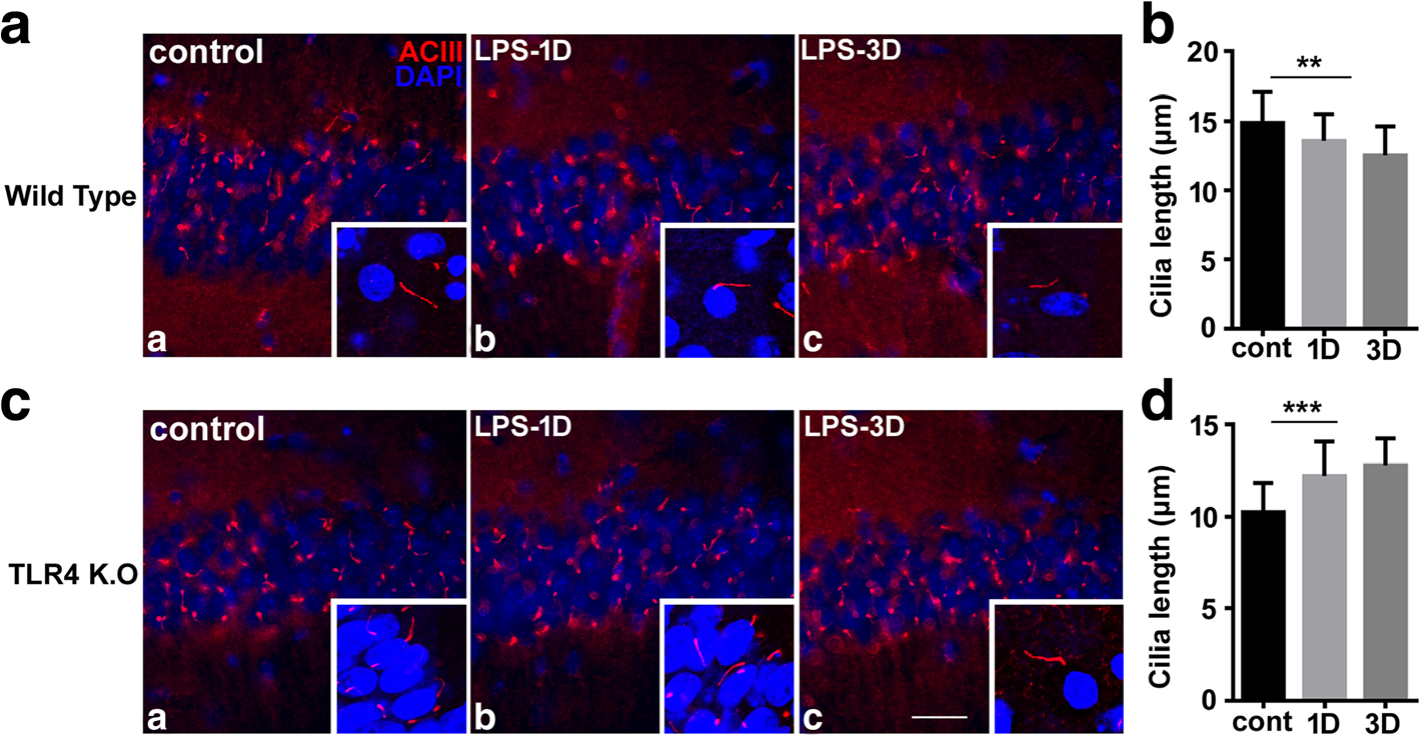
Primary ciliary length decreased in hippocampal pyramidal neurons after LPS treatment in wild type mice, but increased in TLR4^−/−^ mice. **The** brains from wild type (**a**) and TLR4^−/−^ mice (TLR4 K.O (**c)**) were stained for ACIII (red) and DAPI (blue) after intracerebroventricular injection of LPS. Panels show representative images from four to five mice before (control) and 1 (LPS-1D) and 3 (LPS-3D) days after LPS treatment. ACIII-reactive ciliary length (**b**, **d**) in the CA1 region was measured at the same time points. Ciliary length was significantly shorter in wild type mice, but longer in TLR4^−/−^ mice, after LPS treatment. The data are presented as mean ± SEM. **p < 0.01, ***p < 0.001. Scale bar = 20 μm in **a** and **c**; 10 μm in the corresponding inset images

To determine the role of primary cilia in neuroinflammation, we examined changes in hippocampal cilia in a mouse model of inflammation. Because LPS administration induces neuroinflammation, this model is likely suitable for evaluating ciliary function in the hippocampus [19]. To identify cilia, we stained the hippocampus for ACIII, a universal marker of primary cilia in the mouse brain [11]. Whereas staining for somatostatin receptor 3 could potentially be used for the same purpose, this stain labels only approximately 30–50% of ACIII-immunoreactive cilia in neurons [20]. Therefore, ACIII is a useful marker for analyzing ciliary length. In control mice, ACIII was found exclusively in primary cilia. It was especially abundant in the pyramidal layer of the CA1 and CA3 regions of the hippocampus. Intracerebroventricular injection of LPS reduced the length of cilia in the CA1 region of wild type (WT) mice 1 and 3 days post-treatment (Fig. 1a, b).

We next examined the effects of LPS on ciliary length in TLR4^−/−^ mice. Early studies report that TLR4 is expressed primarily in microglia, but not astrocytes or neurons [21]. Other studies, however, have found that neurons do express TLR4 and that TLR signaling in neurons regulates neural precursor cell proliferation, axonal growth, neuronal plasticity, and adult neurogenesis [22]. Because TLR4 is the primary receptor for bacterial LPS, we utilized TLR4^−/−^ mice to evaluate the effects of LPS on primary cilia. Interestingly, basal ACIII expression in the hippocampus of TLR4^−/−^ mice had a mean length of 10.1 μm, which is less than the 15.0 μm measured in WT mice (Fig. 1b, d and Additional file 1: Figure S1 and Additional file 2: Figure S2). Cilia lengths were also measured with images accumulated from 2 μm z-stack in total 24-μm thickness tissues (Additional file 1: Figure S1). In contrast to WT mice, LPS injection into the hippocampus of TLR4^−/−^ mice increased ciliary length in CA1 pyramidal neuronal cells 1 and 3 days post-treatment (Fig. 1c, d). ACIII positive cells were imaged in their entirety at higher magnification with a confocal microscope using 3D reconstruction, and ciliary lengths were analyzed (Additional file 2: Figure S2). These data demonstrate that LPS significantly decreases the length of cilia in the hippocampus, while it increases ciliary length in TLR4^−/−^ mice. Our results suggest that LPS requires interaction with its receptor, TLR4, to stimulate downstream signaling culminating in disruption of ciliogenesis.

### Ciliary ARL13B expression is associated with TLR4-mediated NF-κB activity and decreases in the hippocampus after LPS treatment

**Fig. 2:**
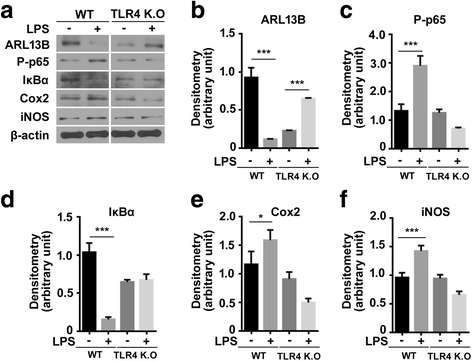
Ciliary ARL13B expression decreased in the hippocampus after LPS treatment and is associated with TLR4. Wild type (WT) and TLR4^−/−^ (TLR4 K.O) mice received intracerebroventricular injections of LPS. Three days after LPS injection, the hippocampus was isolated for immunoblotting (**a**). Expression of ARL13B (**b**), phosphorylated p65 (P-p65) (**c**), IκBα (**d**), Cox2 (**e**), and iNOS (**f**) were quantified

**Fig. 3:**
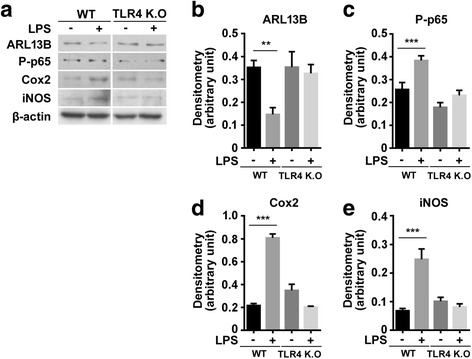
The expression of ciliary ARL13B and inflammatory protein in primary neuronal culture derived from wildtype and TLR4^−/−^ mouse brain. Protein levels of ARL13B (**a**, **b**) and phosphorylation of p65 (**a**, **c**), Cox2 (**a**, **d**), and iNOS (**a**, **e**) were quantified by Western blotting in primary neuronal cells from wild type and TLR^−/−^ mice after LPS. The data are normalized to β-actin and quantified and expressed as optical densities and are presented as the mean ± SEM of three independent experiments. ***p < 0.001

While ACIII has utility in immunohistochemistry as a marker of primary cilia in many regions of the adult mouse brain [11], we have found that another ciliary protein, ARL13B, ciliary trafficking coordinator and universal marker, is more suitable for immunoblotting and immunocytochemistry. Because stimulation of TLR4 activates NF-κB signaling to promote the expression of cytokines, chemokines, and adhesion molecules [23, 24], we next measured the expression of ciliary proteins and indicators of TLR4-mediated NF-κB signaling. In wild type mice, ARL13B protein levels in the hippocampus were decreased after LPS injection (Fig. 2), which is consistent with that of Fig. 1 imaging data. LPS injection increased phosphorylation of p65 and decreased IκBα expression (Fig. 2), indicating translocation of p65 into the nucleus and activation of NF-κB. LPS injection also increased Cox2 and iNOS expression, which are associated with pro-inflammatory cytokines (Fig. 2). In contrast, LPS treatment suppressed NF-κB signaling and expression of pro-inflammatory mediators, and increased ARL13B expression in TLR4^−/−^ mice (Fig. 2). Since LPS has effects on cilia length and ciliary protein in TLR4^−/−^ mice, we confirmed the changes of cilia length with the administration of TLR4 antagonist together with LPS to the wild type mice to determine whether it abrogates LPS-elicited effects on cilia. TLR4 antagonist (1 μg/5 μl, MAb-mTLR4/MD2) was injected 1 day before LPS, and then, ciliary protein and inflammatory mediators were examined. As expected, ARL13B protein level and other inflammatory mediators (p-p65, IKBα, Cox2, and iNOS) showed similar patterns with TLR4^−/−^ mice (Additional file 3: Figure S3). In addition, we measured ciliary protein levels by LPS in primary neuronal culture from wild type and TLR4^−/−^ mice. We confirmed that the expression of ARL13B protein was decreased in wild type, but not changed in TLR4^−/−^. The other inflammatory mediators (p-p65, Cox2, and iNOS) were influenced by LPS in wild type but not in TLR4^−/−^ (Fig. 3). These data suggest that LPS treatment decreases ciliary length (Additional file 1: Figure S1 and Additional file 2: Figure S2) and protein expression in the hippocampus of wild type mice, while LPS increases to some extent in TLR4^−/−^ mice but not to that of the WT mice. Our results suggest that primary cilia may be associated with the LPS-induced TLR4/NF-κB-mediated inflammatory response.

### LPS treatment decreases primary cilia length in hippocampal neurons in vitro

**Fig. 4:**
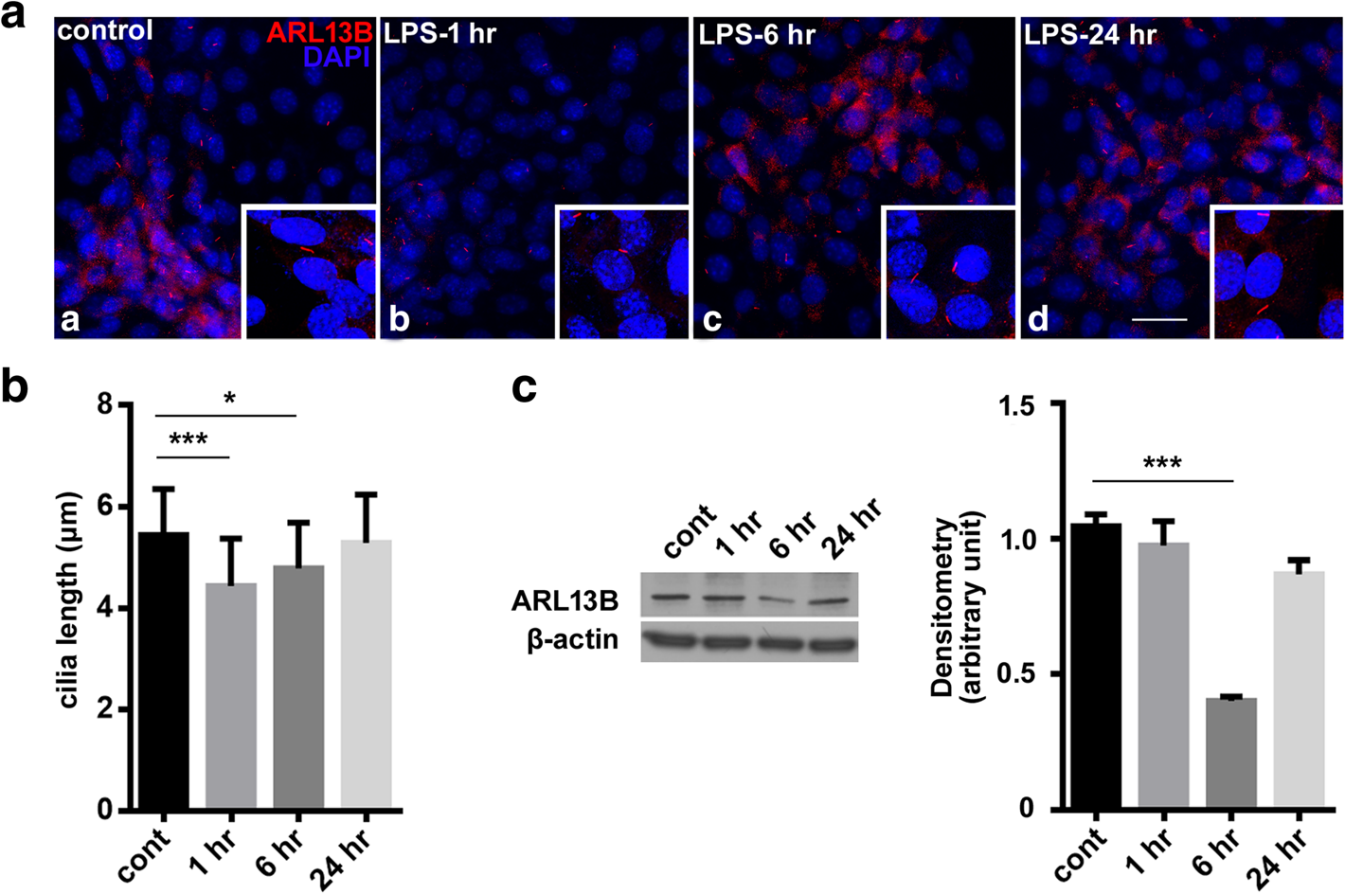
LPS treatment decreased primary ciliary length in cultured hippocampal neurons. After exposure to LPS (100 ng/ml) for 1, 6, and 12 h, HT22 hippocampal neurons were stained (**a**) for ARL13B (red) and DAPI (blue) and ciliary length was measured via confocal imaging (**b**). Over 100 cilia were measured for each group. Protein levels of ARL13B were quantified by Western blotting (**c**) under control conditions (cont) and after LPS exposure. The data are normalized to β-actin and quantified and expressed as optical densities and are presented as the mean ± SEM of three independent experiments. ***p < 0.001. Scale bar = 20 μm in **a**; 10 μm in the corresponding inset images

Constitutive expression of TLR4 has been detected in hippocampal neurons [25], sensory neurons [26], and neural stem cells [27]. These observations suggest that neurons can synthesize chemokines, as well as the cytokines tumor necrosis factor alpha and IL-6 in response to bacterial infection and LPS. In the preceding experiments, we found that ciliary length decreased in hippocampal neurons after LPS treatment and that these changes were altered in TLR4^−/−^ mice. TLR4, however, is also expressed by microglia, and TLR4 activation in microglia mediates inflammation in response to central nervous system infection [28]. We, therefore, sought to determine whether the changes we observed in the hippocampus were due to binding of LPS to TLR4 in neurons or microglia. We thus utilized HT22 hippocampal neuronal cells to exclude the effects of microglial TLR4 activation. In monolayer cultures, freshly isolated HT22 cells had primary cilia with a mean length of 5.5 μm. Treatment with LPS (100 ng/ml) significantly decreased the length of cilia in HT22 cells after 1 and 6 h of exposure, with recovery after 24 h of exposure (Fig. 4a, b). This effect was accompanied by a decrease in ARL13B expression, which reached a nadir after 6 h of exposure (Fig. 4c, d). These data further confirm that LPS-induced inflammation abrogates primary ciliary elongation and protein expression in hippocampal neuronal cells.

### LPS-induced NF-κB signaling and expression of inflammatory mediators are modulated by primary cilia

Primary cilia are present in neurons, mediate mechanical and chemical signal transduction, and contain proteins that support intraciliary transport and structure [8, 9]. Kinesin-2 family protein A (Kif3a), a kinesin motor subunit, is required for proper ciliary function and structure [29, 30]. Kinesin-2 forms a heterotrimeric complex consisting of the motor subunits, Kif3a and Kif3b, and a non-motor subunit, kinesin-associated protein [31]. Kinesin-2 motor proteins mediate anterograde transport to the distal tip of the cilium, while dynein motor proteins mediate retrograde transport to the basal body of the cilium [32].

**Fig. 5:**
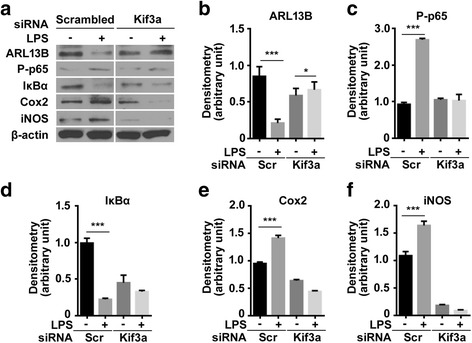
LPS-induced NF-κB signaling and expression of inflammatory mediators are associated with primary cilia. HT22 cells were transfected with control (scrambled) siRNA or siRNA targeting Kif3a for 24 h before exposure to LPS (100 ng/ml). Six hours after LPS treatment, cells were harvested and stained for detection of ARL13B (**a**, **b**), phosphorylated p65 (P-p65) (**a**, **c**), IκBα (**a**, **d**), Cox2 (**a**, **e**), and iNOS (**a**, **f**). The data are normalized to β-actin and quantified and expressed as optical densities and are presented as the mean ± SEM of three independent experiments. *p < 0.05, ***p < 0.001

We utilized knockdown of Kif3a with siRNA to assess whether LPS-induced NF-κB signaling and expression of inflammatory mediators are influenced by primary ciliary function. Twenty-four hours after transfection with Kif3a siRNA, HT22 hippocampal cells were exposed to LPS (100 ng/ml) for 6 h. In control cells, LPS decreased ARL13B and IκBα expression and increased phosphorylation of p65, indicating nuclear translocation of p65 and activation of NF-κB signaling (Fig. 5a–d). LPS treatment also increased Cox2 and iNOS expression (Fig. 5a, e, and f). In Kif3a-silenced cells, basal ARL13B expression was decreased compared with scrambled siRNA-treated cells (Fig. 5a, b). LPS increased ARL13B expression, but it did not reach basal control levels in scrambled siRNA-treated cells (Fig. 5a, b). Those immunoblotting data was confirmed with ARL13B length with image analysis (Additional file 4: Figure S4). LPS treatment after Kif3a knockdown did not affect phosphorylation of p65 and IκBα expression, indicating suppression of NF-κB signaling. Basal expression of Cox2 and iNOS was inhibited with Kif3a knockdown and was further decreased by LPS (Fig. 5c–f).

Next, we measured the levels of pro- and anti-inflammatory cytokines. The levels of inflammatory cytokines [tumor necrosis factor (TNF)α, interleukin (IL)-6, and IFN-γ] were attenuated in under Kif3a siRNA treated cells, while anti-inflammatory cytokines [IL-10] was unchanged (Additional file 5: Figure S5). We also observed that the LPS treatment increased the nitric oxide (NO) production, but this effect was attenuated in Kif3a knockdowned cells (Additional file 6: Figure S6). Finally, we detected the effects of primary cilia on acetylcholinesterase (AChE) in hippocampal neuronal cells since it is reported that TLR-mediated cholinergic reactions determine the inflammatory outcome in traumatized mice and humans [33]. The results showed that the level of AChE increased after LPS treatment. However, LPS treatment in Kif3a knockdowned cells did not alter the level compared to scrambled siRNA-treated cells (Additional file 7: Figure S7).

On the other hand, previous study showed IL-1β-induced cilia elongation with alternations to cilia trafficking of ARL13B [14]. The elongation is associated with a transient increase in HIF-2α expression and accumulation in the primary cilium. To clarify cilia trafficking in neuroinflammation, we examined HIF-2α expression level. The protein level of HIF-2α was increased in HT22 neuronal cells after LPS, but it was abrogated in Kif3a-silenced cells (Additional file 8: Figure S8). To exclude the effect of LPS on proliferation, we examined the cell proliferation in our condition. Ki67 expression by LPS was not changed compared to both scrambled- and Kif3a-silenced cells (Additional file 8: Figure S8). This suggested that LPS does not impact in cell proliferation, which potentially serve as a direct regulator of ciliogenesis and thereby influence cilia prevalence and length. These data suggest that primary cilia inhibit LPS-induced NF-κB activation and inhibit the expression of pro-inflammatory mediators.

## Discussion

In this study, we found that LPS-induced inflammation decreases the length of cilia in hippocampal pyramidal neuronal cells. Alternations in ciliary length were associated with TLR4-mediated NF-κB activation and expression of pro-inflammatory cytokines; these processes appear to be modulated by ciliary function.

The biogenesis of primary cilia was investigated to track the stages of primary cilia assembly [17]. In this study, we used immunostaining with universal ciliary markers ACIII and ARL13B for measuring ciliogenesis. As shown in the “Results,” basal ACIII imaging of the pyramidal neurons hippocampus of the WT and TLR4^−/−^ resulted in cilia mean length of 15.0 and 10.1 μm, respectively (Fig. 1b, d). However, the basal mean length as measured via ARL13B was 5.8 μm (Fig. 4b). This discrepancy in the basal lengths highlights the current limitations of the ciliogenesis assay, mainly in that it cannot monitor the ciliary stages in real time. It also points to the current challenges in measuring the absolute cilia lengths. Thus, we focused on comparing the differences of their relative cilia lengths and on the effects of primary cilia in neuroinflammatory conditions.

Primary cilia have well-established roles in the regulation of many signaling pathways [34]. There are, however, fewer studies linking cilia to NF-κB activation and the release of inflammatory cytokines, although there is a growing body of evidence for the case [12–14]. The present study itself demonstrates that NF-κB activation and expression of pro-inflammatory cytokines are associated with ciliary function. As another example, removal of the cilium inhibits LPS-induced expression of Cox2 and iNOS due to failure in upstream signaling rather than in transcription. Recent studies have also directly linked primary cilia to IL-1β-induced NF-κB signaling through the regulation of IKK activity [12]. Specifically, traffic in the cilium appears to be important to phosphorylation of IκB by IKK, which initiates degradation of IκB. Heat shock protein 27 (hsp27), an established regulator of IKK, is also localized to the ciliary axoneme, and cellular levels of hsp27 are dramatically perturbed with loss of the primary cilium. These findings suggest that primary cilia exert potentially critical modulation of NF-κB activity. Indeed, it has been proposed by others that cilia can modulate molecular events at rate-limiting steps of the NF-κB signaling pathway to fine-tune signaling as appropriate [12]. Our present data support this hypothesis.

On the other hand, recent insights identified cholinergic anti-inflammatory pathway to play an important role in the process of reducing inflammation [35–37]. As we found the effects of primary cilia on neuroinflammation, we examined the relation of cholinergic pathway and primary cilia. While AChE level was increased after LPS treatment, there was no significant difference in its level between the Kif3a knockdowned cells compared to the scrambled siRNA-treated control cells (Additional file 7: Figure S7). This data suggested that the primary cilia may have little relationship with the cholinergic pathway.

With regard to human disorders, primary ciliary dyskinesia (PCD) is a genetic disorder involving dysfunction of motile cilia, which share much in structural homology with primary cilia [38]. Low nitric oxide (NO) is a clinical marker of PCD, and there is evidence that cells isolated from patients with PCD have a reduced capacity to respond to infectious challenge [39]. In that study, ciliated cell cultures from patients with PCD did not increase NO production and *NOS2* expression remained unchanged. Those findings suggest that primary cilia contribute to NO production, which involves activation of NF-κB. Similarly, human disorders in which primary ciliary dysfunction has been implicated, including Jeune syndrome and short rib-polydactyly syndrome type III, have been recently linked to WDR34, which is itself associated with NF-κB signaling [40, 41]. It has also been reported that IKK/NF-κB-induced inflammation interferes with ependymal ciliogenesis [42] and primary cilia disassembly in stem cells [43]. Collectively, available evidence suggests that primary cilia may regulate NF-κB-induced inflammation, in addition to integrating extracellular signaling pathways and establishing cell polarity, which in turn determine neuronal cell fate, migration, differentiation, and multiple adult behaviors [34].

In this study, we found that TLR4-mediated NF-κB activation and expression of pro-inflammatory cytokines are modulated by ciliary function. Differential and receptor-specific TLR expression on ciliated/basal cells or on the apical/basolateral cell membrane of the airway epithelium has been documented [44]. It has also been reported that LPS-induced intracellular sub-apical TLR4 expression in human primary polarized bronchial epithelial cells is associated with cilia on the apical cell surface [45]. These studies reveal that LPS-induced activation of respiratory epithelial cells is largely dependent on TLR4 signaling in motile cilia, suggesting that LPS and its binding partner, TLR4, are linked to cilia. Other studies show that nasal NO production, ciliary beat frequency, and TLR signaling are impaired in pulmonary non-tuberculous mycobacterial disease [46]. In the present study, we report that ciliary length is shorter in TLR4^−/−^ mice compared with control and that LPS injection into the hippocampus increased the length of cilia in CA1 pyramidal neurons.

Theoretically, LPS is the most potent TLR4 agonist, which implies that LPS should not stimulate the neuronal cells of TLR4^−/−^ mice. In our study, LPS increased ciliary length in TLR4^−/−^ mice, but increased length did not reach basal levels of wild type. While the mechanism of this slightly increased ciliary length under LPS is currently unclear, it may be resulted from existence of other LPS receptors, such as TLR2, nucleotide oligomerization domain (Nod)-like receptors (NLRs) [47], and cannabinoid receptor [48]. However, it is obvious that primary cilia or ciliary proteins (e.g., HSP27, Kif3a) modulate inflammatory response by LPS because the ARL13B protein is lower in TLR4^−/−^ mice compared to wild type and inflammatory response (cytokine, NF-κB activation) is dependent on Kif3a-knockdowned neuronal cells. More detailed studies on ciliary protein trafficking with NF-κB activity remains to be pursued.

Taken together, our data suggest that TLR4 activation by LPS is associated with ciliary function. The presence and length of cilia are dependent upon various factors, including the expression and activities of ciliary proteins [49], inflammation [13], and mechanical stress [50]. The primary cilium already has known important roles in inflammation. Here, we suggest for the first time that the cilium is also of fundamental importance in neuroinflammation. The ciliogenesis or expression of the cilia provides a potential therapeutic targets for neuroinflammatory conditions.

## Conclusions

Our data revealed that ciliary function could modulate TLR4-mediated NF-κB activation and expression of pro-inflammatory cytokines. These data were supported by shorter ciliary length in TLR4^−/−^ mice compared with control and by the increased length of cilia in CA1 pyramidal neurons after LPS injection into the hippocampus. All these data strongly suggest that TLR4 activation in hippocampal neurons is associated with ciliary function. For the first time, this study revealed novel aspects about the relationship between primary cilia, TLR4 signaling, and NF-κB activity in hippocampal neurons. Our data highlight the need to investigate the contributions of ciliary proteins to NF-κB activity or neuroinflammation in greater detail.

## Additional files

Additional file 1: Figure S1. Panorama images from confocal microscopy in wild type and TLR4^−/−^ mice. The brains from wild type and TLR4^−/−^ mice were stained for ACIII. Confocal imaging was taken with 2 μm z-stack in total 24-μm thickness hippocampal tissues and was reconstructed to 3D image (XZ plane) with × 40 water immersion lens. (TIFF 289 kb)

Additional file 2: Figure S2. High resolution with a confocal microscope using 3D reconstruction in wild type and TLR4^−/−^ mice after LPS or not. ACIII-positive cells were imaged in their entirety at higher magnification with a confocal microscope using 3D reconstruction (XYZ plane, 2 μm z-stack in total 24-μm thickness hippocampal tissues), and ciliary lengths were analyzed in wild type and TLR4^−/−^ mice after LPS treatment. (TIFF 942 kb)

Additional file 3: Figure S3. The expression of ciliary ARL13B and inflammatory protein after TLR4 antagonist followed by LPS in wild type mice. TLR4 antagonist (1 μg/5 μl, MAb-mTLR4/MD2, Invivogen) was intracerebroventricularly injected 1 day before LPS, and then, ciliary protein and inflammatory mediators were examined. The data are quantified and expressed as optical densities and are presented as the mean ± SEM of three independent experiments. *p < 0.05, **p < 0.01, ***p < 0.001. (TIFF 1467 kb)

Additional file 4: Figure S4. Image analysis for ciliary length in ARL13B stained cilia in Kif3a knockdowned hippocampal neuronal cells after LPS treatment. HT22 cells were transfected with control (scrambled) siRNA or siRNA targeting Kif3a for 24 h before exposure to LPS (100 ng/ml). Six hours after LPS treatment, cells were harvested and stained for detection of ARL13B. The data are presented as mean ± SEM ***p < 0.001. Scale bar = 20 μm (TIFF 2534 kb)

Additional file 5: Figure S5. Analysis of inflammatory cytokines in Kif3a knockdowned hippocampal neuronal cells after LPS treatment. Inflammatory cytokines also were increased in the Kif3a knockdowned hippocampal neuronal cells after LPS according to manufacturer’s protocols. One-way analysis of variance (ANOVA); all data are shown as mean ± SD, where ***p < 0.001 denotes a significant difference compared with the control group. (TIFF 258 kb)

Additional file 6: Figure S6. Measurement of nitric oxide (NO) production in Kif3a knockdowned hippocampal neuronal cells after LPS treatment. NO production was measured according to manufacturer’s protocols. LPS treatment increased NO production, but the effect was reversed in Kif3a knockdowned cells. The data are the representative of three experiments. All the data are shown as mean ± SD, where ***p < 0.001 denotes a significant difference compared with the control group. (TIFF 178 kb)

Additional file 7: Figure S7. Effects of primary cilia on the levels of AchE in Kif3a knockdowned hippocampal neuronal cells after LPS treatment. The AChE substrate in the kits was incubated with neuronal cells homogenates. Quantification of thiocholine reflects the ACheE activities. Note that there was no significance with scrambled and Kif3a knockdowned cells after LPS treatment. (TIFF 207 kb)

Additional file 8: Figure S8. The expression of HIF2α, Ki67, and Kif3a in Kif3a knockdowned hippocampal neuronal cells after LPS treatment. Protein levels of HIF2α, Ki67, and Kif3a were quantified by Western blotting. (TIFF 455 kb)

## Abbreviations

ACIII: Acetylcholine III
ARL13B: ADP ribosylation factor like GTPase 13B
Cox2: Cyclooxygenase 2
DG: Dentate gyrus
IGF: Insulin growth factor
IL-1β: Interleukin-1β
iNOS: Inducible nitric oxide synthase
LPS: Lipopolysaccharide
Shh: Sonic hedgehog
TGF: Transforming growth factor
TLR4: Toll-like receptor
WT: Wild type
