# Supplementary figures and images for: Primary cilia modulate TLR4-mediated inflammatory responses in hippocampal neurons
Source: J Neuroinflammation. 2017 Sep 19;14:189. doi: 10.1186/s12974-017-0958-7 (PMC5606072; doi:10.1186/s12974-017-0958-7)

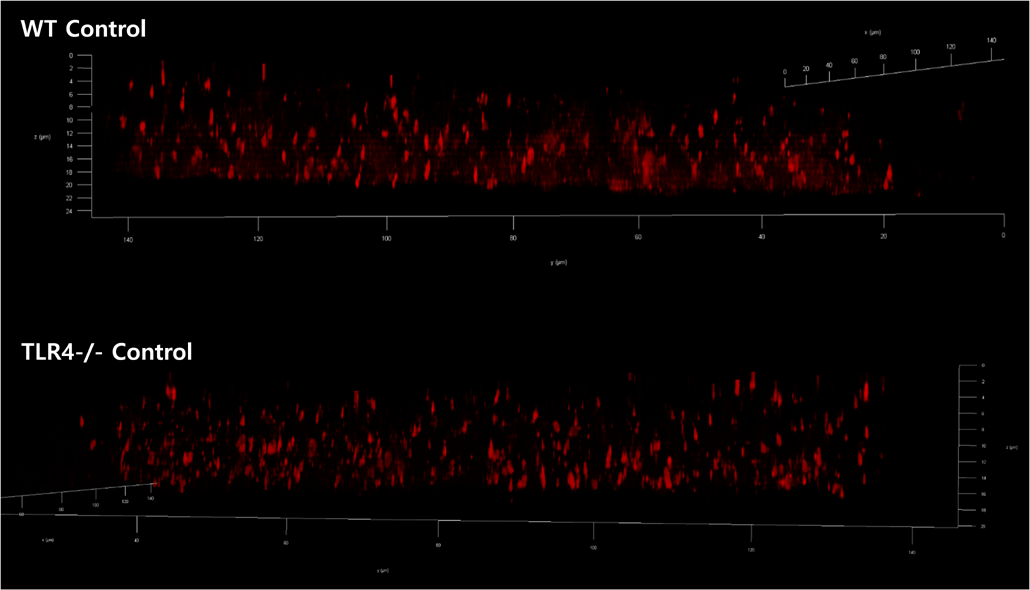

Supplement: Supplementary file 1 — Panorama images from confocal microscopy in wild type and TLR4−/− mice. The brains from wild type and TLR4−/− mice were stained for ACIII. Confocal imaging was taken with 2 μm z-stack in total 24-μm thickness hippocampal tissues and was reconstructed to 3D image (XZ plane) with × 40 water immersion lens. (TIFF 289 kb) [file 12974_2017_958_MOESM1_ESM.tif]

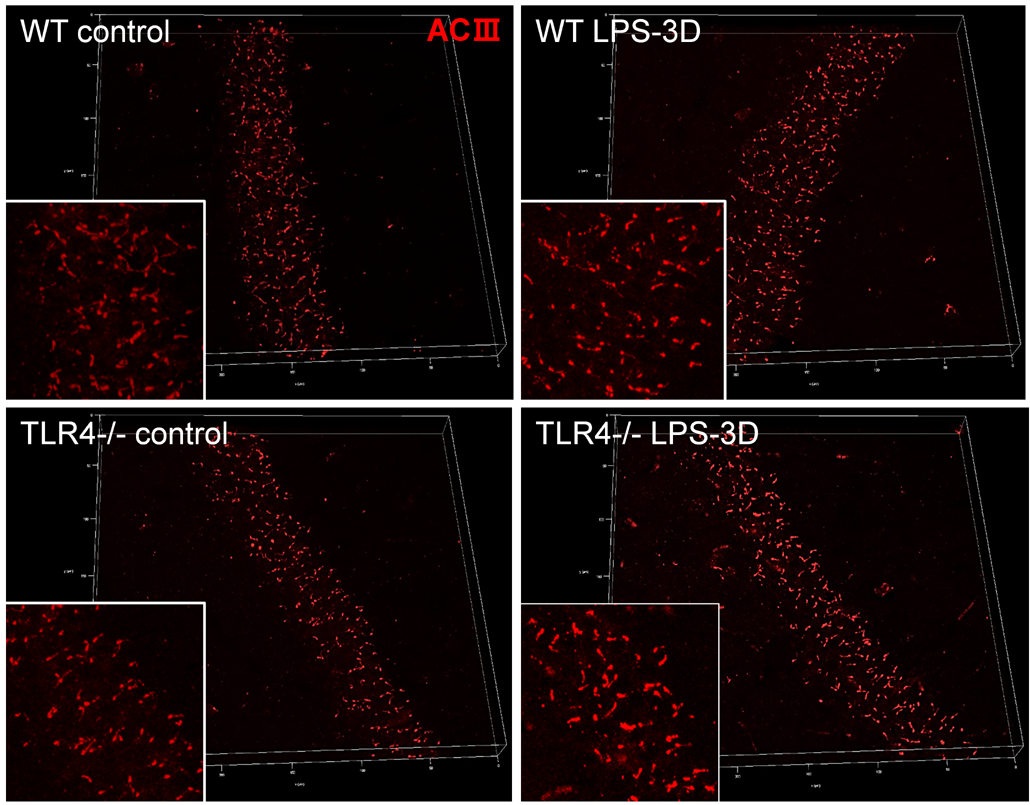

Supplement: Supplementary file 2 — High resolution with a confocal microscope using 3D reconstruction in wild type and TLR4−/− mice after LPS or not. ACIII-positive cells were imaged in their entirety at higher magnification with a confocal microscope using 3D reconstruction (XYZ plane, 2 μm z-stack in total 24-μm thickness hippocampal tissues), and ciliary lengths were analyzed in wild type and TLR4−/− mice after LPS treatment. (TIFF 942 kb) [file 12974_2017_958_MOESM2_ESM.tif]

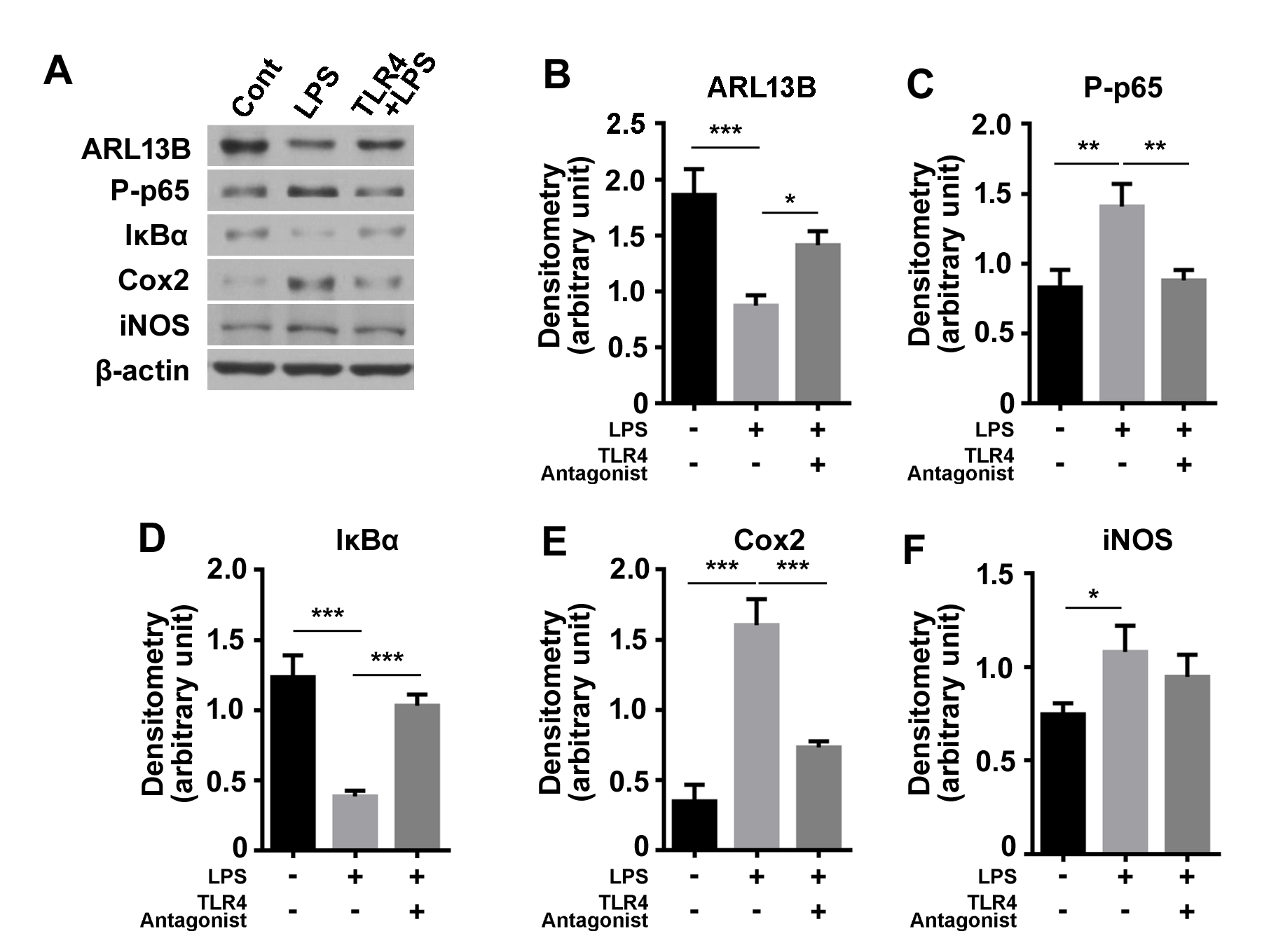

Supplement: Supplementary file 3 — The expression of ciliary ARL13B and inflammatory protein after TLR4 antagonist followed by LPS in wild type mice. TLR4 antagonist (1 μg/5 μl, MAb-mTLR4/MD2, Invivogen) was intracerebroventricularly injected 1 day before LPS, and then, ciliary protein and inflammatory mediators were examined. The data are quantified and expressed as optical densities and are presented as the mean ± SEM of three independent experiments. *p < 0.05, **p < 0.01, ***p < 0.001. (TIFF 1467 kb) [file 12974_2017_958_MOESM3_ESM.tif]

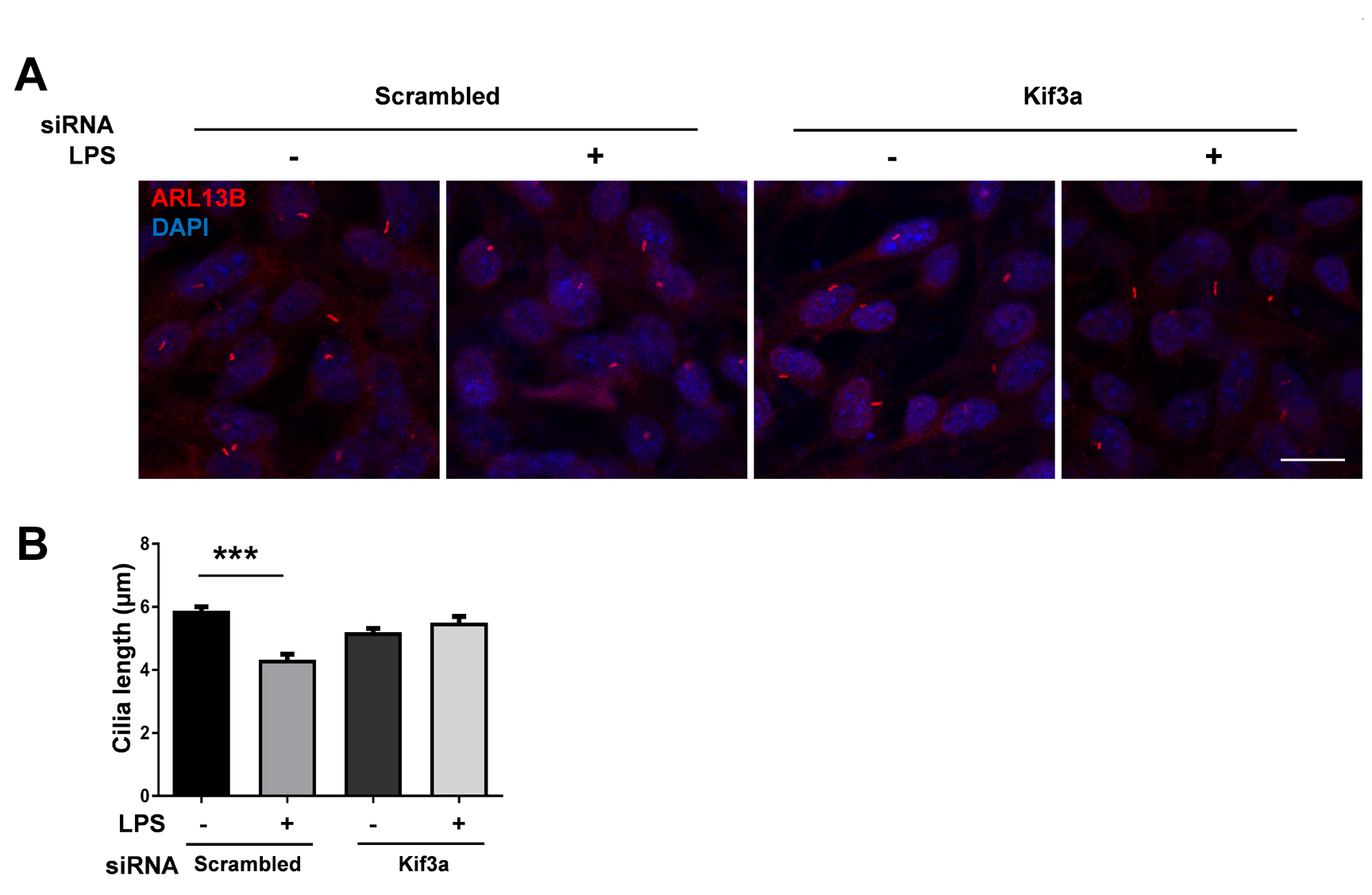

Supplement: Supplementary file 4 — Image analysis for ciliary length in ARL13B stained cilia in Kif3a knockdowned hippocampal neuronal cells after LPS treatment. HT22 cells were transfected with control (scrambled) siRNA or siRNA targeting Kif3a for 24 h before exposure to LPS (100 ng/ml). Six hours after LPS treatment, cells were harvested and stained for detection of ARL13B. The data are presented as mean ± SEM ***p < 0.001. Scale bar = 20 μm (TIFF 2534 kb) [file 12974_2017_958_MOESM4_ESM.tif]

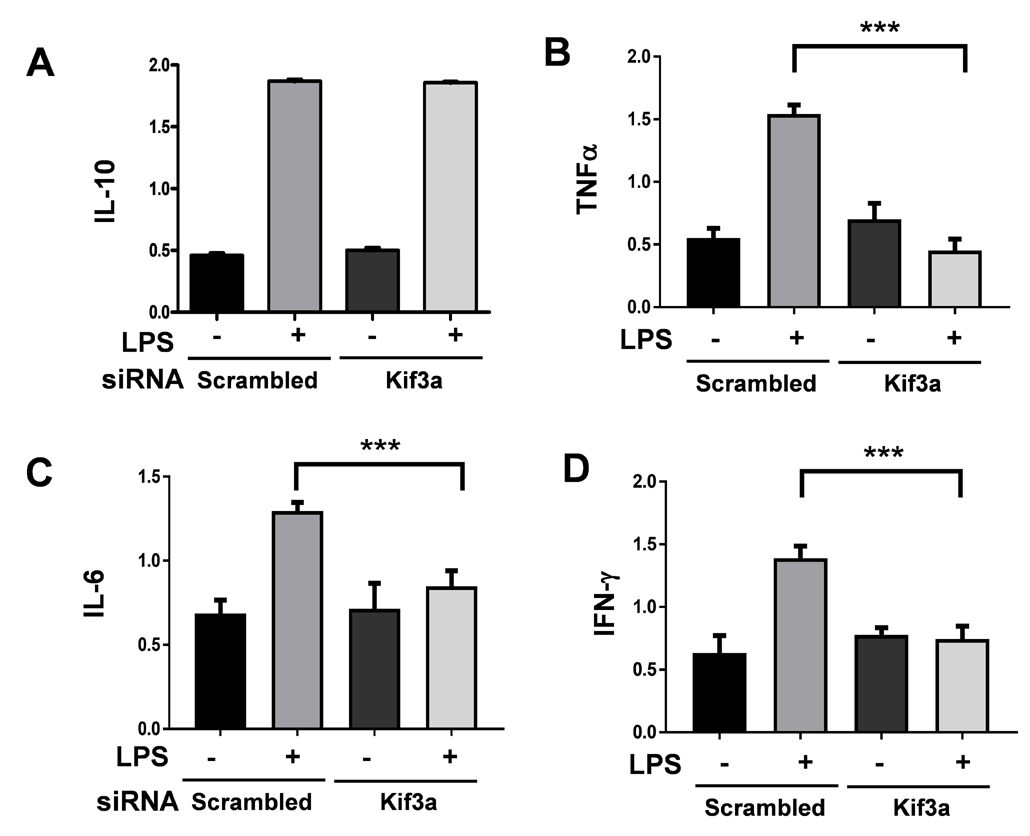

Supplement: Supplementary file 5 — Analysis of inflammatory cytokines in Kif3a knockdowned hippocampal neuronal cells after LPS treatment. Inflammatory cytokines also were increased in the Kif3a knockdowned hippocampal neuronal cells after LPS according to manufacturer’s protocols. One-way analysis of variance (ANOVA); all data are shown as mean ± SD, where ***p < 0.001 denotes a significant difference compared with the control group. (TIFF 258 kb) [file 12974_2017_958_MOESM5_ESM.tif]

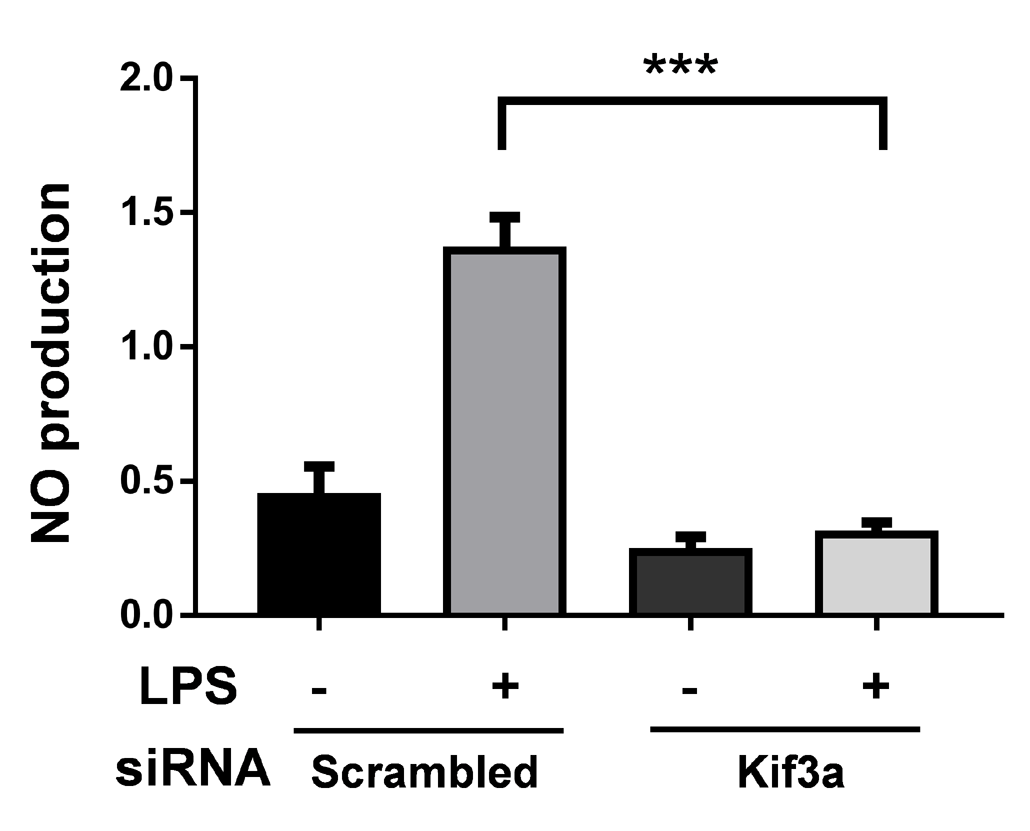

Supplement: Supplementary file 6 — Measurement of nitric oxide (NO) production in Kif3a knockdowned hippocampal neuronal cells after LPS treatment. NO production was measured according to manufacturer’s protocols. LPS treatment increased NO production, but the effect was reversed in Kif3a knockdowned cells. The data are the representative of three experiments. All the data are shown as mean ± SD, where ***p < 0.001 denotes a significant difference compared with the control group. (TIFF 178 kb) [file 12974_2017_958_MOESM6_ESM.tif]

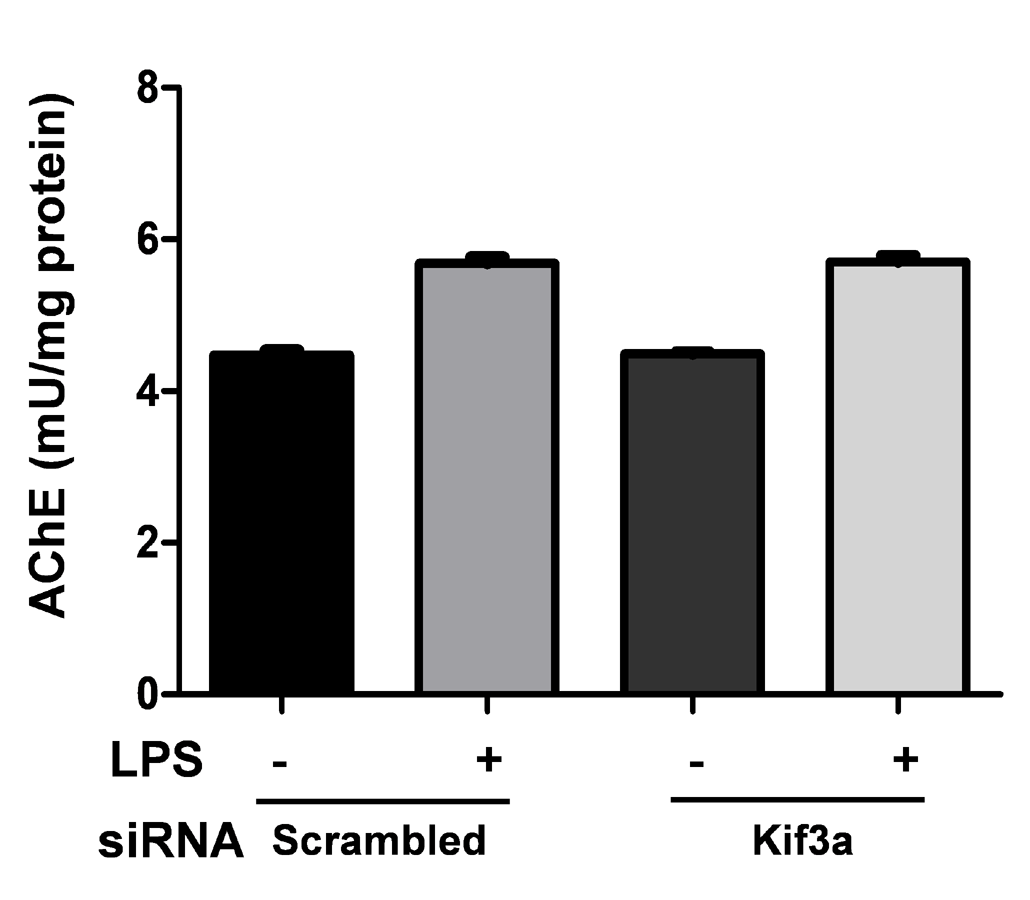

Supplement: Supplementary file 7 — Effects of primary cilia on the levels of AchE in Kif3a knockdowned hippocampal neuronal cells after LPS treatment. The AChE substrate in the kits was incubated with neuronal cells homogenates. Quantification of thiocholine reflects the ACheE activities. Note that there was no significance with scrambled and Kif3a knockdowned cells after LPS treatment. (TIFF 207 kb) [file 12974_2017_958_MOESM7_ESM.tif]

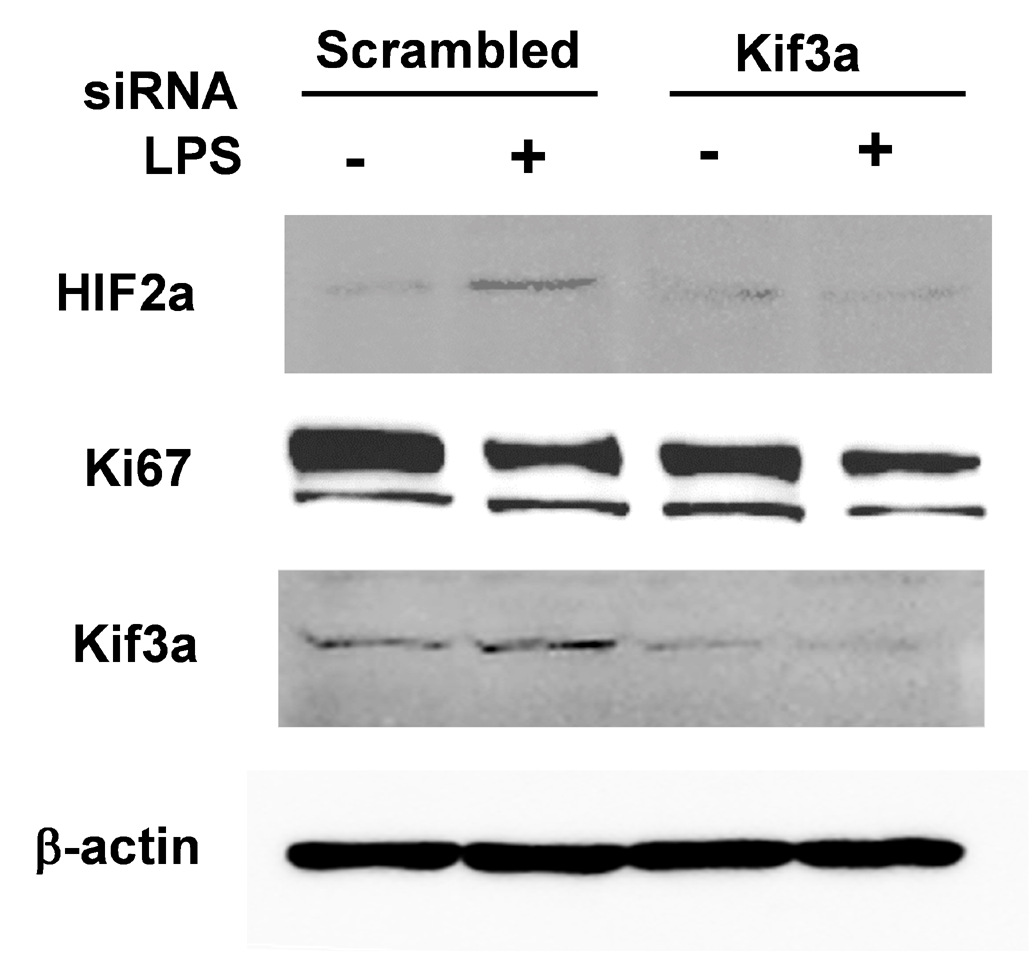

Supplement: Supplementary file 8 — The expression of HIF2α, Ki67, and Kif3a in Kif3a knockdowned hippocampal neuronal cells after LPS treatment. Protein levels of HIF2α, Ki67, and Kif3a were quantified by Western blotting. (TIFF 455 kb) [file 12974_2017_958_MOESM8_ESM.tif]
